
JOURNAL INFORMATION
==============================
NLM Title Abbreviation: Gastroenterol Rep (Oxf)
Journal ID: Gastroenterol Rep (Oxf)
NLM Journal ID: 101620508
Journal ID: gastro

Gastroenterology Report

EISSN: 2052-0034
Publisher: Oxford University Press

ARTICLE INFORMATION
==============================
PMCID: PMC5554385
DOI: 10.1093/gastro/gox032
Article ID: gox032
Article version: 1
Subjects: Chinese Abstracts

Chinese Abstracts – Volume 5 Issue 3*

* This article abstract was originally published in English. This translation has not been verified and should not be relied upon—it is provided for reference purposes only. The Publishers have not checked this translation and accept no liability for completeness or accuracy of this translation or the use of this translation for whatever purpose. This translation may be incomplete and inaccurate in whole or in part. If you need to rely upon a translation of this abstract, a professional human translator should be engaged to supply an accurate translation of the original English. When referencing articles from this journal, please always refer to the original English version, rather than a translated equivalent.

Print publication date: 2017 Aug
Electronic publication date: 2017 Aug 11
Volume: 5
Issue: 3
First page: C1
Last page: C3
Copyright: © The Author(s) 2017. Published by Oxford University Press and Sixth Affiliated Hospital of Sun Yat-Sen University
Copyright year: 2017
License: This is an Open Access article distributed under the terms of the Creative Commons Attribution License (http://creativecommons.org/licenses/by/4.0/), which permits unrestricted reuse, distribution, and reproduction in any medium, provided the original work is properly cited.
License URL: https://creativecommons.org/licenses/by/4.0/

Page count: 3

==============================
OXFORD

*Gastroenterology Report*, 2017, 1–3

doi: 10.1093/gastro/gox032

ABSTRACTS

### Chinese Abstracts – Volume 5 Issue 3*

### Review（综述）

### Perioperative use of immunosuppressive medications in patients with Crohn's disease in the biological era
### 生物治疗时代下免疫抑制药物在克罗恩病患者围手术期的应用

[摘要] 克罗恩病（CD）是胃肠道透壁性炎症性疾病，可导致炎症、狭窄和/或瘘。一旦患者疾病顽固药物治疗症状无缓解， 或出现机械性肠梗阻、 瘘或穿孔时，则需行外科手术治疗。遗憾的是，绝大多数情况下手术并不是根治性的， 因此， 应当重视对肠道的保留和围手术期药物治疗。由于许多 CD 治疗药物都是免疫抑制性的，从而导致了人们对术后感染性并发症及吻合口愈合的担心。但对药物治疗的这担忧应该与其预防和治疗残留或复发疾病所带来的获益相权衡。我们对相关文献进行了综述， 并对生物治疗时代下免疫抑制药物在克罗恩病患者术前、围手术期及术后的应用进行了一些推荐。制定围手术期药物治疗的标准化路径将有助于今后进一步针对提高外科疗效和预防疾病复发进行研究。

关键词：克罗恩病；生物治疗；围手术期；药物治疗

### Systematic review of mixed cryoglobulinemia associated with hepatitis E virus infection: association or causation?
### 戊肝病毒感染相关的混合性冷球蛋白血症的系统综述：相关关系还是因果关系？

[摘要] 背景与目的：混合性冷球蛋白血症（MC）已显示与一些病毒感染有关，其中慢性丙型肝炎被认为是 MC 发生一个主要原因。有研究报道了戊肝病毒（HEV）相关的 MC，但对于这种罕见的关系所知甚少。本研究的目的是对 HEV 相关的 MC 进行系统综述，评估两者之间是否存在因果关系。方法：由一位经验丰富的图书馆情况员按照先验标准， 对每一个数据库截至 2016 年 12 月 12 日的文献进行检索。评估偏倚风险；采用 Hill 标准判断因果关系。结果： 5 项研究满足纳入标准，共计 15 例病例。偏倚风险评估结果显示，3项研究为低风险，1项中低风险，1 项中等风险。入组病例中位年龄 43 岁，所有病例都来自西欧。 2 例患者免疫功能正常， 13例免疫抑制， 系实体器官移植后同时合并慢性戊型肝炎。 7 例患者出现肾脏病变，其中 1 例为轻中度冷球蛋白血症，3例为重度冷球蛋白血症。 1 例患者自行改善，1例患者在在减少免疫抵制剂的剂量后 HEV 得以清除。 10 例患者接受聚乙二醇干扰素或病毒唑治疗 3 个月，所有病例冷球蛋白血症消失且均获治疗结束病毒学应答，但仅 2 例患者获得持续病毒学应答。 3 例患者接受免疫抑制剂治疗后，冷球蛋白血症均消失。最终，1 例患者出现慢性肾功能衰竭，3例患者发展为终末期肾病，1 例患者死亡。因果关系分析显示，9项 Hill 标准满足 5 项。结论： MC 与 HEV 感染有关，且 HEV 与 MC 之间很可能存在一个因果关系。

关键词：戊肝病毒；肝炎；病毒学；混合性冷球蛋白血症；系统综述

### Variceal bleeding in cirrhotic patients
### 肝硬化静脉曲张出血

[摘要] 静脉曲张出血是肝硬化患者死亡的一个主要原因。对于其急性期的处理及二级预防都已明确。最近的 2015 Baveno VI专家共识认为，肝硬化静脉曲张出血应作为一个急症，在肝脏或普通 ICU 中接受治疗。其治疗策略包括早期应用血管活性药物（内镜治疗前）、预防性抗生素及限制性输血方案（目标血红蛋白是 7 g/dL）。内镜治疗主要是血管结扎术，而硬化疗法已被摒弃。对于一些严重的肝病患者（Child Pugh C级或初始内镜治疗时发现活动性出血的 Child Pugh B 级），应在入院后 72 h 内行经颈静脉肝内门体分流术（TIPS）， 以降低再出血的风险。二级预防主要是联合应用非选择性 β 受体阻滞剂（NSBBs）和重复的血管结扎术。当无法耐受 NSBBs 二级预防、或尽管很好地接受了二级预防但仍然发生再次出血时，抑或是对于顽固性出血，应考虑行 TIPS 。而当 TIPS 无法控制出血或对于肝功能已严重恶化的病例，可考虑肝移植。

关键词：静脉曲张出血；肝硬化；内镜治疗；非选择性 β 受体阻滞剂；行经颈静脉肝内门体分流术；肝移植

### Original article（论著）

### Selection of a cut-off for high- and low-methane producers using a spot-methane breath test: results from a large North American dataset of hydrogen, methane and carbon dioxide measurements in breath
### 单次甲烷呼气试验判断甲烷浓度高低的阈值选择：来自一个大型北美氢气-甲烷-二氧化碳呼气试验数据库的结果

[摘要] 背景：甲烷-氢气呼气试验，是在加入碳水化合物底物后，每 15–20 min 间隔收集一次呼气标本，共 10 个样本，对这 10 个样本中甲烷和氢气浓度进行检测。采用该法判断氢气浓度的升高需要频繁的样本检测，而且氢气浓度升高 otepad 多发生于呼气试验后期；相较之下，甲烷浓度

*This article abstract was originally published in English. This translation has not been verified and should not be relied upon—it is provided for reference purposes only. The Publishers have not checked this translation and accept no liability for completeness or accuracy of this translation or the use of this translation for whatever purpose. This translation may be incomplete and inaccurate in whole or in part. If you need to rely upon a translation of this abstract, a professional human translator should be engaged to supply an accurate translation of the original English. When referencing articles from this journal, please always refer to the original English version, rather than a translated equivalent.
本文摘要最初是以英文发表。中文摘译未获认证，谨供参考。出版社没有对中文摘译进行核验，不负责译文的完整性和准确性，也不会将其用以其他目的。中文摘译可能会出现部分不完整或不准确的情况。如果你需要准确获悉摘要，最好能请一位专业译者针对英文原文进行翻译。阅读杂志文章，请参考英文原文，不推荐中文翻译。

一般在底物加入前即可升高。如果将甲烷浓度作为呼气试验的主要目标,单次甲烷检测(例如收集禁食一晚后单个时间点的呼气样本, 无需加入底物)或许足矣。

方法:我们分析了11,674 例连续检测的十样本乳酸呼气试验结果,这些病例来自于过去一年美国各州,所有呼气标本均在 Commonwealth 实验室(Salem, MA, USA)进行检测。将北美共识指南(NAC)的呼气试验标准作为参考标准。

结果:参照 NAC 标准, 该数据库病例的甲烷总体阳性率粗略为18.9%, 校正后为 20.4%。判断甲烷浓度高低的最佳阈值为≥ 4 ppm, 其灵敏度为94.5%(可信区间:93.5%~95.4%),特异度为 95.0%(可信区间:94.6%~95.5%)。采用校正因子(以 5%的$CO_2$ 作为分子)进行校正后,有 0.7% 的高甲烷浓度重新判定为低浓度,2.1%的低甲烷浓度被判定为高浓度。

结论:无论是基于本数据库所得出的≥ 4 ppm 还是另一项单中心研究所描述的 ≥ 5 ppm 作为呼气试验中甲烷基线浓度的阈值,用于判断传统十样本乳酸呼气试验检测小肠细菌过度生长时所诊断的"甲烷阳性",都具有很高的准确性。

关键词:呼气试验;单次甲烷呼气试验;诊断试验;小肠细菌过度生长

### Fecal microbiota transplantation for *Clostridium difficile* infection in patients with ileal pouches
### 粪菌移植治疗回肠储袋患者艰难梭菌感染

[摘要] 背景:回肠储袋肛管吻合患者中艰难梭菌感染(CDI)越来越多地见诸报道。本研究目的是评估粪菌移植(FMT)对 CDI 储袋患者的治疗效果。

方法:从我们 IRB 批准的前瞻性储袋疾病数据库中选取 2012 — 2016 年间所有因 CDI 行 FMT 的病例。主要结局指标是 FMT 治疗后粪便检查艰难梭状芽胞杆菌(*C. difficile*)阴性率,次要结局指标是症状缓解情况和内镜下病变改善情况。

结果:共 13 例患者纳入研究,其中 10 例(76.9%)为白人男性。所有病例均因溃疡性结肠炎行 J 型储袋手术。在经过平均(2.8 ± 0.8)个疗程的抗生素治疗失败后,共施行了 22 次 FMT 治疗,平均每例患者(1.7 ± 1.1)次。在 22 次治疗中,16 次是通过储袋镜、4 次通过食管胃十二指肠镜、2 次通过灌肠进行 FMT 治疗。在初次 FMT 治疗后,所有病例*C. difficile* PCR检测均为阴性;58.3%(7/12)的病例症状得到缓解;根据修订储袋疾病活动指数,27.3%(3/11)的病例内镜下病变获得改善。在(1.2 ± 1.1)年的随访期间,5例(38.5%)患者在初次 FMT 治疗后复发,其中 4 例成功接受了再次 FMT 治疗。

结论: FMT 似乎能有效治疗回肠储袋患者的 CDI 。然而,FMT对内镜下炎症病变的改善作用有限,且治疗后容易复发。

关键词:艰难梭状芽胞杆菌;粪菌移植;回肠储袋;储袋炎

### Real world experience with Pegylated Interferon and ribavirin in Hepatitis C Genotype 1 population with favourable *IL28B* polymorphism

[摘要] 背景与目的:丙型肝炎(HCV)的传统治疗是应用聚乙二醇干扰素(FEG-IFN)和病毒唑,但副作用明显。*IL28B*基因多态性可以预测治疗反应,CC基因型者效果更好。*ITPA* 基因缺失能对治疗引起的临床贫血起到保护作用。本研究目的是检测接受FEG-IFN和病毒唑治疗的 1 型 HCV 患者中 *IL28B* 基因多态性和 *ITPA* 基因突变情况,并分析其与持续病毒学应答(SVR)的相关性。

方法:通过病历记录查询过去 10 年所有接受 FEG-IFN 和病毒唑治疗的 1 型 HCV 患者,通过信函邀请其参加本次研究。采集患者血液标本用以 *IL28B* 和 *ITPA* 基因检测。从病历记录中提取治疗反应、贫血发生及治疗中断情况。

结果:共有 61 例 1 型 HCV 患者接受了 FEG-IFN 和病毒唑治疗,其中 42 例同意参加本次研究。治疗时平均年龄为(45.6 ± 12.9)岁,男性患者占 83.3%。 33 例(78.6%)检测为 *IL28B* CC基因型,其中 25 例 (75.8%)获得SVR,而 9 例非 CC 基因型患者中仅 3 例(33.3%)获得 SVR ($P$=0.041)。 11 例患者检测为*ITPA* AC基因型,30例为 CC 基因型。*ITPA* AC与 CC 基因型患者的临床贫血发生率的差异无统计学意义(45.5% *vs*. 63.3%, $P$=0.302)。即便在出现临床贫血的患者中,仍有70.8%最终获得了 SVR 。治疗中断对 SVR 亦无影响。

结论:对于*IL28B* CC基因型的 1 型 HCV 患者,在考虑新的治疗选择前,应告知其FEG-IFN和病毒唑具有良好的治疗效果。

关键词:丙型肝炎;聚乙二醇干扰素;*IL28B*基因多态性;持续病毒学应答

### Healthcare utilization and costs associated with cholangiocarcinoma
### 胆管癌相关的医疗情况及治疗费用

[摘要] 背景:胆管癌是一种罕见但致命的恶性肿瘤,预后差,病死率高。随着近年来胆管癌诊治水平的提高, 胆管癌在美国的发病率和治疗效果是否得到了改善尚不清楚。本研究的目的是以美国住院患者为对象, 评估胆管癌发病率、治疗费用及住院病死率的变化趋势。

方法:收集美国住院样本数据库(NIS) 1997 — 2012 年间第一出院诊断为胆管癌(ICD-9代码:155.1和 156)的患者。采用趋势回归分析评估研究期间住院人数、住院时间、住院费用以及住院病死率的时间趋势。

结果:研究期间,胆管癌作为主要诊断的住院人数显著增加,从 1997 年的10,357增加至 2012 年的 11,970($P$<0.001)。胆管癌患者的平均住院时间缩短了 17%, 从 1997 年的9.5天缩短至 2012 年的 7.9 天($P$<0.001)。然而,在此期间,每例患者的平均住院费用(按通货膨胀进行调整后)增高了113.25%,由 1997 年的36,460美元提高至 77,753美元。住院病死率由 1997 年的 9.3%下降至 2012 年的6.4%($P$<0.001)。

结论: 1997 — 2012 年间,美国胆管癌住院人数和人均住院费用显著增加,而住院病死率则明显下降。

关键词:胆管癌;住院人数;住院费用;住院病死率;流行病学

### Identification of the distinguishing features of Crohn's disease and ischemic colitis using computed tomographic enterography
### 用以鉴别诊断克罗恩病与缺血性结肠炎的 CT 小肠造影影像学特征

[摘要] 背景与目的:由于克罗恩病(CD)与缺血性结肠炎(ISC)的临床处理不同,因此对这两种疾病的鉴别诊断是非常重要的。 ISC 易被误诊为 CD,尤其是对于老年人群。这两种疾病各自的影像学特征尚未被发明了。本研究的目的是评估 CT 小肠造影(CTE)用于 CD 与 ISC 鉴别诊断的价值。

方法:通过Cleveland Clinic Foundation的电子医疗记录,检索确诊为 CD 和 ISC 的病例。入组其中接受过 CTE 检查者,无论其是否同时接受了肠镜和组织病理检查。由一名胃肠影像学专家盲法重新阅读 CTE 图像。计算每一项 CTE 影像学特征鉴别 CD 与 ISC 的灵敏度、特异度、准确性、阳性预测值及阴性预测值。采用 Kappa 系数($\kappa$)来评估 CTE 与参考标准的诊断一致性。

结果:共计 34 例患者纳入研究,其中 CD 17例,ISC 17例。对 CD 与 ISC 进行鉴别诊断,CTE图像中黏膜强化和"靶征"缺失诊断 CD 的灵敏度均为 100%, 但这两种影像学特征的特异度较低, 分别为35.3%和76.5%。CTE 图像中的狭窄诊断 CD 的灵敏度仅为64.7%,但其特异度高达100%。黏膜强化、狭窄、"靶征"缺失鉴别 CD 与 ISC 的准确性分别为67.7%、82.4%和 88.2%。联合应用黏膜强化和"靶征"缺失,其鉴别诊断的准确性可达 100%。在鉴别 CD 与 ISC 方面,CTE与参考标准具有较好的一致性($\kappa$=0.882)。

结论:在鉴别 CD 与 ISC 方面,CTE似乎具有较高的临床价值。

关键词: CT 小肠造影;克罗恩病;缺血性结肠炎;鉴别诊断

### Rectal cancer restaging using 3D CUBE *vs*. 2D T2-weighted technique after neoadjuvant therapy: a diagnostic test
### 3D CUBE与2D T2加权成像技术应用于直肠癌新辅助治疗后再分期的比较:一项诊断试验

[摘要] 目的 本研究旨在比较 3D CUBE 序列与 2D T2加权快速自旋回波(FSE)序列用于直肠癌新辅助治疗后再分期的价值。

方法：本回顾性研究纳入了 72 例经肠镜和病理活检证实的直肠癌患者。新辅助治疗后， 所有病例接受盆腔磁共振成像（MRI）检查， 1.5T MRI 扫描序列包括层厚 1.4 mm 的冠状位 3D CUBE T2 加权 FSE 序列和层厚为 5 mm 的矢状位、冠状位及轴位 2D T2 加权 FSE 序列。记录两种序列总的收集时间。以术后病理为金标准， 评估两种成像技术的再分期准确性。绘制受试者操作特征（ROC）曲线并计算曲线下面积（AUC）。

结果：阅片人一判断 3D T2 加权成像和 2D T2 加权成像对 T 分期的诊断准确率分别为 81.9% 和 72.2%, 阅片人二分别为 86.1% 和 75.0%。判断肿瘤是否突破肌层时, 3D 序列的 AUC 明显高于 2D 序列（阅片人一：0.878 *vs.* 0.783；阅片人二：0.905 *vs.* 0.796；均$P<0.05$）。而在判断是否有淋巴结转移时，两种序列 AUC 的差异无统计学意义（阅片人一：0.719 *vs.*0.698；阅片人二：0.740 *vs.* 0.698, 均 $P>0.05$）。在肠壁显示度、肿瘤显示度和总体图像质量方面， 两种序列的差异均无统计学意义（均 $P>0.05$）。但相较于 2D 序列, 3D 序列收集时间更短, 信号强度比率更高（均 $P<0.05$）。

结论：相较于 2D T2 加权 FSE 序列, 3D CUBE T2 加权序列能更准确地对新辅助治疗后的直肠癌进行再分期, 而且其扫描时间更短, 并能进行多方位的图像重建。

关键词：直肠癌；磁共振成像；3D CUBE；新辅助治疗；再分期；图像质量

### Ammonia level at admission predicts in-hospital mortality for patients with alcoholic hepatitis
### 入院时血氨水平可预测酒精性肝炎患者的住院病死率

[摘要] 目的：酒精性肝炎（AH）是慢性或急性饮酒者一种特有的临床综合征, 具有较高的短期死亡率。在急性肝衰竭患者中, 血氨浓度升高者病死率增高；然而, 其对 AH 患者的影响尚不明确。

方法：在一家三甲医院中，对出院诊断为 AH 的患者进行回顾性分析。将满足 AH 诊断标准的病例纳入最终的数据分析。建立多因素逻辑回归模型用以评估血氨水平对住院病死率和 30 天病死率的预测价值。并对其中的肝性脑病患者进行亚组分析。

结果：在 105 例纳入研究的 AH 患者中, 26例（25%）在首次住院期间死亡。在 79 例出院患者中, 30例（39%）于 30 天内死亡。 82 例患者入院时进行了血氨检测, 其中, 住院期间死亡的 25 例患者血氨水平明显增高 （97 *vs.* 69 μmol/L, $P<0.01$）。而在 57 例出院患者中, 30天内死亡与未死亡病例血氨水平的差异无统计学意义（71 *vs.* 67 μmol/L, $P=0.69$）。将血氨水平加入包括年龄、性别、肝硬化、治疗方式、终末期肝病模型（MELD）评分的多因素回归模型后， 模型预测住院病死率的 C 值由 0.708 显著提高至 0.801, 预测术后 30 天病死率的 C 值则由 0.756 轻微升高至 0.766。针对肝性脑病患者进行亚组分析, 亦得到了类似的结果。

结论： AH 患者入院时血氨水平升高，其住院死亡风险增加；而当患者顺利出院后，血氨水平升高似乎并不会影响 30 天病死率。

关键词：酒精性肝炎；肝性脑病；氨；病死率

### Case report（病例报告）

### Pancreatitis of ectopic pancreatic tissue: a rare cause of gastric outlet obstruction
### 异位胰腺组织的胰腺炎：胃出口梗阻的罕见原因

### Wandering spleen: a surgical enigma
### 游走脾：手术之谜

### A diagnostic dilemma: acute abdomen presenting as segmental arterial mediolysis masked by a ruptured hepatocellular carcinoma
### 诊断困境：节段性动脉中层溶解引起的腹痛被肝细胞癌破裂所掩盖
